# Post-operative day one discharge following craniotomy for tumour resection

**DOI:** 10.1007/s11060-026-05790-2

**Published:** 2026-09-23

**Authors:** Krishmila Yogeswaran, Frederick Ewbank, Rebecca Khoo, Daniel O’flaherty, Md Islam, Antony Kaldas, Arif Zafar, Andrew Durnford, Paul Grundy, Joy Roach

**Affiliations:** 1 Department of Neurosurgery, University Hospital Southampton NHS Foundation Trust, Tremona Road, Southampton, Hampshire SO16 6YD UK; 2 University of Southampton, Southampton, Hampshire UK

**Keywords:** Brain tumour, Craniotomy for tumour resection, Early discharge, Neuro-oncology, Post-operative day 1 discharge, Readmission

## Abstract

**Purpose:**

Post-operative admission following craniotomy for tumour resection (CTR) remains standard practice. To improve efficiency, optimise patient flow, and reduce length of stay (LOS), a post-operative day one (POD1) discharge pathway was implemented. This study evaluated the feasibility of POD1 discharge and identified factors associated with delayed discharge and 30-day unplanned readmission.

**Methods:**

A retrospective analysis of 263 consecutive patients undergoing CTR between November 2022 and December 2023 was performed. Baseline demographic, clinical, tumour-related, operative, and post-operative variables were collected. The primary outcome was POD1 discharge. Secondary outcomes included 30-day unplanned readmission and factors associated with discharge and readmission. Univariable logistic regression and Fisher’s exact test were used.

**Results:**

POD1 discharge was achieved in 146 patients (55.5%). The overall 30-day unplanned readmission rate was 8.0%, including 7.5% among patients discharged on POD1. Mean cumulative 30-day hospital days were 1.0 days among patients discharged on POD1 compared with 7.0 days among patients requiring prolonged admission. POD1 discharge was not associated with increased odds of readmission (OR 0.87, 95% CI 0.35–2.17, *p* = 0.760). Diabetes mellitus (OR 0.41, 95% CI 0.17–0.96, *p* = 0.044), post-operative adverse events (OR 0.18, 95% CI 0.06–0.47, *p* = 0.001), and post-operative neurological deficits (OR 0.05, 95% CI 0.01–0.17, *p* < 0.001) were associated with reduced odds of POD1 discharge. Eloquent tumour location was associated with increased odds of readmission (OR 3.35, 95% CI 1.36–8.78, *p* = 0.010).

**Conclusion:**

POD1 discharge following CTR was feasible in more than half of patients and was not associated with increased 30-day readmission. These findings support structured POD1 discharge pathways in appropriately selected patients.

## Purpose

Patients undergoing craniotomy for tumour resection (CTR) are traditionally admitted for several days following surgery to facilitate post-operative neurological monitoring and recovery. However, shorter post-operative length of stay (LOS) is increasingly recognised as an important indicator of high-quality surgical care, reflecting efficient recovery, optimised healthcare resource utilisation, improved patient flow, and reduced healthcare expenditure. In neuro-oncology, early discharge may additionally support patient-centred care by facilitating recovery within the home environment, particularly for patients with life-limiting diagnoses.

Enhanced Recovery After Surgery (ERAS) pathways have demonstrated that LOS may be reduced across multiple surgical specialties without compromising patient safety [1–3]. Within neurosurgery, emerging evidence suggests that accelerated discharge following CTR may be feasible in selected patients, particularly as many post-operative complications occur within the immediate post-operative period rather than several days after surgery [4]. Previous studies have identified factors including good pre-operative functional status as predictors of post-operative day one (POD1) discharge following brain tumour resection [5–7]. Despite these findings, POD1 discharge following CTR remains uncommon in routine neurosurgical practice globally.

The Department of Neurosurgery at University Hospital Southampton NHS Foundation Trust (UHS) has utilised an early discharge strategy since 2006. Following encouraging findings from an initial cohort of same day discharges, a formalised POD1 discharge pathway was implemented and expanded, prompting the present study [8]. This study aimed to evaluate the feasibility of POD1 discharge following CTR and to identify factors associated with delayed discharge and 30-day unplanned readmission.

## Methods

263 consecutive neuro-oncology patients who underwent CTR were identified retrospectively from a prospective neurosurgery database at UHS between November 2022 and December 2023.

### Protocol

Administrative and logistic reasons were contributors to an extended LOS in existing literature [9]. Patients were prospectively assessed for suitability for POD1 discharge as part of routine peri-operative care. Factors favouring POD1 discharge included good baseline functional status, absence of major medical frailty, anticipated uncomplicated post-operative recovery, and ability to mobilise safely following surgery. Final discharge eligibility required completion of post-operative MRI, physiotherapy and occupational therapy clearance, a stable neurological examination, and medical fitness for discharge.

Patients were discharged with a printed discharge summary, an information leaflet on discharge instructions following craniotomy as well as verbal discharge instructions from ward nursing staff. Follow-up was conducted through telephone clinic review after availability of histopathological results. Patients were additionally provided with contact details for the neuro-oncology clinical nurse specialist as their first point of contact for post-discharge concerns.

### Study design

Patient data was obtained retrospectively from the Trust’s electronic medical record system. To ensure comprehensive capture of readmissions, including admissions occurring outside the primary centre, the online referral system (https://www.referapatient.org) was utilised. Where necessary, local hospitals were contacted directly via telephone to confirm admission status and LOS, ensuring complete ascertainment of 30-day readmissions across the regional network.

Demographic variables collected included age, sex, and address. Clinical variables included primary cancer diagnosis, common comorbidities (specifically respiratory, cardiovascular and diabetes mellitus), use of pre-operative corticosteroids in patients with diabetes, history of pre-operative seizures and pre-operative World Health Organisation (WHO) Performance Status. Tumour- related factors like location, involvement of eloquent brain regions, histopathological diagnosis was collected. Extent of tumour resection on post-operative MRI was also recorded. Operative and peri-operative variables included duration of surgery, awake versus asleep, adverse events, and post-operative neurological deficits. Adverse events were defined as post-operative complications requiring additional investigation, treatment, or monitoring during the index admission. Post-operative neurological deficits were defined as new or worsened neurological impairments identified following surgery, including motor, language, sensory, visual, or cognitive deficits. Neurological deficits were recorded and analysed separately from adverse events.

Finally, data on admission and discharge dates, and length of stay (LOS) were collected. 30-day unplanned readmission and receipt of adjuvant oncological treatment were additionally recorded.

### Outcomes

The primary outcome was post-operative day one (POD1) discharge, defined as discharge on the first post-operative day following surgery. Secondary outcomes included 30-day unplanned readmission, cumulative LOS and factors associated with prolonged admission and delayed discharge. Cumulative 30-day hospital days were calculated as the length of the index admission plus any subsequent readmission days occurring within 30 days of discharge.

### Statistical analysis

Fisher’s exact test was run to determine the association between POD 1 discharge and 30-day readmission as well as corticosteroid use and its association to discharge timing due to a low event frequency. Univariable logistic regression analysis was performed to estimate odds ratios (OR) for the association between individual variables and a POD1 discharge and 30-day unplanned readmission.

Where data were unavailable, analyses were performed using available-case methodology. Results are presented as frequency and percentage for patients who achieved a POD1 and for those who were readmitted within 30 days. Regression results are reported as OR with corresponding 95% confidence intervals (CI). Statistical significance was defined as *p* < 0.05. Where estimates could not be generated due to inadequate subgroup size, these values were not reported.

Data was recorded and managed using ‘Microsoft Excel’ (Version 2508 Build 16.0.19127.20648). Statistical analysis was performed using ‘R’ software (version 4.5.3 (2026-03-11 ucrt).

## Results

### Feasibility of POD1 discharge outcome

**Fig. 1:**
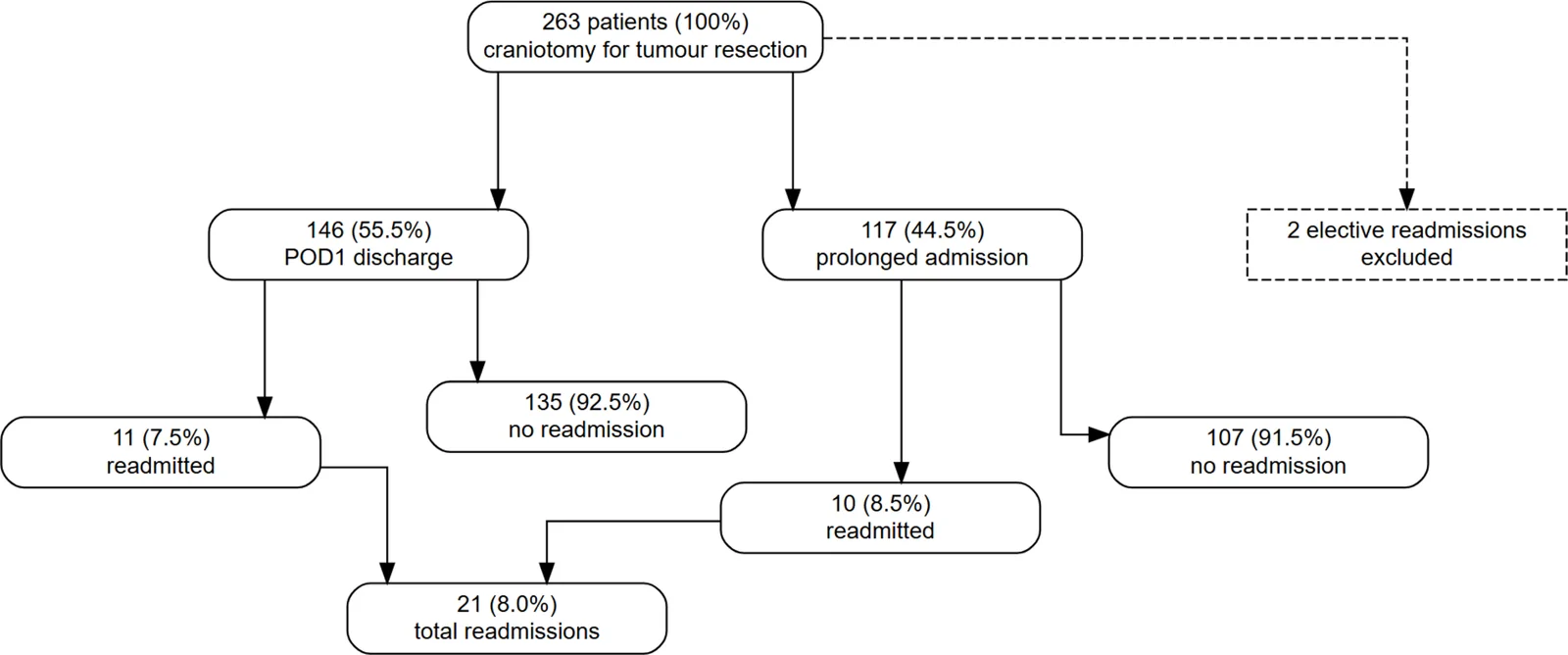
Study flow diagram. Flowchart demonstrating patient inclusion and exclusion within the study cohort and subsequent POD1 discharge and readmission outcomes

A total of 263 patients underwent craniotomy for tumour resection during the study period. Of these, 146 patients (55.5%) were discharged on post-operative day one (POD1). Overall, 21 patients (8.0%) underwent unplanned readmission within 30 days of discharge. Among patients discharged on POD1, 11 (7.5%) experienced unplanned readmission within 30 days. Two patients who underwent elective readmission were excluded from the readmission analysis (Fig. 1).

**Fig. 2:**
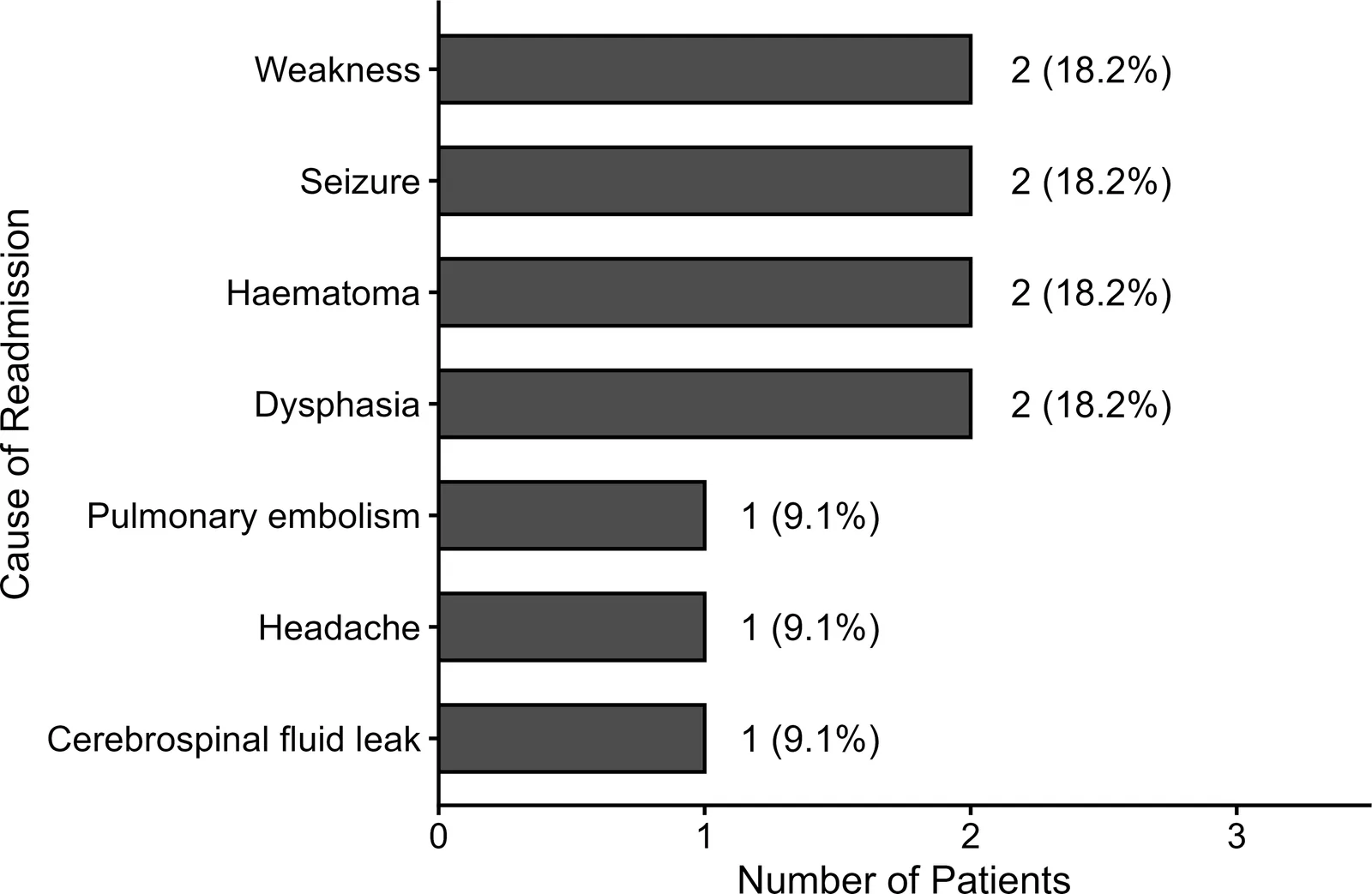
Causes for 30-day readmission following POD1 discharge. Bar chart depicting causes of unplanned readmission among patients discharged on POD1

There was no significant association between POD1 discharge and 30-day readmission (OR 0.87, 95% CI 0.35–2.17, *p* = 0.760, Fisher’s exact test). Post-operative MRI was completed within 24 h in 258 of 263 patients (98.1%). Delayed MRI acquisition occurred in five patients, four of whom had a LOS of 2 days and one a LOS of 4 days. The mean time to readmission was 9.9 days for those discharged on POD1 and 9.1 days across all readmissions. The mean LOS during readmission was 5.4 days for patients who had POD1 discharge, and 8.0 days for all unplanned readmissions. The causes of readmission amongst patients discharged on POD1 are illustrated in Fig. 2, with the most frequent causes including neurological deficits (weakness or dysphasia), seizures, and post-operative haematoma.

Cumulative 30-day hospital days, defined as the length of the index admission plus any subsequent readmission days within 30 days of discharge, were also evaluated. Mean cumulative 30-day hospital days were 1.0 day (range 1–24 days) among patients discharged on POD1 compared with 7.0 days (range 2–57 days) among patients requiring prolonged admission.

### POD 1 discharge outcome

There was no statistically significant association between age and POD1 discharge (OR 0.99, 95% CI 0.98–1.01, *p* = 0.523). The oldest POD1 discharged patient was aged 82 years and the mean age of this subgroup was 57.4 years. Factors associated with POD1 discharge and their OR, 95% CI and p-values are summarised in Table 1. Baseline clinical characteristics, including primary cancer diagnosis, pre-operative WHO Performance Status, pre-operative seizures and common medical comorbidities, did not demonstrate a significant association with POD1 discharge. Diabetes mellitus was the only pre-operative clinical factor significantly associated with reduced odds of POD1 discharge. Among the 25 patients with diabetes mellitus, corticosteroid use was not associated with discharge timing (OR 1.24, 95% CI 0.18–8.87, *p* = 1.000).

Primary tumour diagnosis, anatomical tumour location and most histological subtypes were not significantly associated with POD1 discharge. Higher WHO tumour grade and IDH wildtype status were associated with increased likelihood of POD1 discharge. Eloquent tumour location and achievement of gross total resection were not significantly associated with discharge outcomes. Out of 25 posterior fossa tumours, 9 achieved POD1 discharge, whereas 16 patients required prolonged admission, with an average LOS of 8 days.

Post-operative adverse events and neurological deficits demonstrated significant associations with failure to achieve POD1 discharge. Patients requiring prolonged admission had a mean LOS of 6.1 days (95% CI 4.66–7.48), with LOS ranging from 2 to 57 days. Among recorded post-operative neurological deficits, dysphasia was the most common, occurring in 10 patients (8.6% of the prolonged-admission cohort). Delayed post-operative MRI was uncommon, occurring in only five patients (1.9%). In the absence of MRI delay, adverse events, or post-operative neurological deficits, prolonged admission was attributable to patients not yet meeting physiotherapy and occupational therapy discharge criteria.

**Table 1:** Factors associated with POD1 discharge

Variable	POD1 Discharge *n* (%) (*n* = 146)	Prolonged Admission *n* (%) (*n* = 117)	OR	95% CI	*p*-value
Clinical Factors					
WHO Performance Status					
WHO 2–3	3 (2.3%)	5 (4.3%)	0.45	0.09–1.90	0.289
Comorbidities					
Respiratory	28 (19.2%)	28 (23.9%)	0.75	0.42–1.37	0.350
Cardiac	35 (24.0%)	36 (30.8%)	0.71	0.41–1.23	0.218
Diabetes	9 (6.2%)	16 (13.7%)	0.41	0.17–0.96	0.044
Pre-operative Seizures	49 (33.6%)	40 (34.2%)	0.97	0.58–1.63	0.915
WHO Grade					
1	17 (17.2%)	34 (29.1%)	Ref	-	-
2	19 (19.2%)	21 (18.0%)	1.81	0.78–4.28	0.172
3	15 (15.2%)	10 (8.6%)	3.00	1.13–8.29	0.030
4	47 (47.5%)	22 (18.8%)	4.27	2.00-9.43	< 0.001
Not specified	1 (1.0%)	0 (0.0%)	-	-	-
Tumour Related Factors					
IDH					
Mutant	18 (27.7%)	16 (13.7%)	Ref	-	-
Wildtype	47 (72.3%)	15 (12.8%)	2.79	1.15–6.88	0.024
Eloquent location					
No	89 (61%)	82 (70.1%)	Ref	-	-
Yes	57 (39%)	35 (29.9%)	1.5	0.90–2.53	0.124
Operative Factors					
Gross total resection					
Yes	97 (66.4%)	64 (54.7%)	Ref	-	-
No	49 (33.6%)	55 (45.3%)	0.61	0.37–1.01	0.053
Anaesthetic					
General	110 (75.3%)	97 (82.9%)	Ref	-	-
Sedation	36 (24.7%)	20 (17.1%)	1.59	0.86–2.92	0.138
Post-operative Factors					
Adverse event					
No	141 (96.6%)	98 (83.8%)	Ref	-	-
Yes	5 (3.4%)	19 (16.2%)	0.18	0.06–0.47	0.001
Post-Operative Neurological Deficit					
No	144 (98.6%)	91 (77.8%)	Ref	-	-
Yes	2 (1.4%)	26 (22.2%)	0.05	0.01–0.17	< 0.001

POD1 discharge n=146; prolonged admission n=117

OR = odds ratio; CI = confidence interval; Ref = reference category; WHO = World Health Organisation

Odds ratios, confidence intervals and p-values are derived from univariable logistic regression analyses and relate to the POD1 discharge outcome

Percentages for IDH status are calculated from patients with available molecular data

Post-operative adverse events and neurological deficits were analysed as determinants of discharge timing rather than pre-operative predictors

### Unplanned readmission within 30 days outcome

Age was not significantly associated with 30-day readmission (OR 1.01, 95% CI 0.98–1.05, *p* = 0.418). The mean age of patients who underwent readmission was 60.23 years. Factors associated with 30-day unplanned readmission and their respective OR, 95% CI and p-values are presented in Table 2. Most baseline demographic, clinical, tumour-related and operative variables were not significantly associated with readmission. Eloquent tumour location was the only factor significantly associated with increased odds of readmission (OR 3.35, 95% CI 1.36–8.78, *p* = 0.010).

**Table 2:** Factors associated with 30-day unplanned readmission

Variable	Readmitted within 30 Days n (%) (*n* = 21)	Not Readmitted within 30 Days *n*(%) (*n* = 240)	OR	95% CI	*p*-value
**Clinical Factors**					
**WHO Performance Status**					
WHO 2–3	1 (4.8%)	7 (2.9%)	1.87	0.10-11.45	0.571
**Comorbidities**					
Respiratory	4 (19.0%)	52 (21.7%)	0.86	0.24–2.44	0.793
Cardiac	7 (33.3%)	63 (26.3%)	1.39	0.51–3.50	0.497
Diabetes Mellitus	3 (14.3%)	22 (9.2%)	1.67	0.37–5.43	0.441
Pre-operative Seizures	4 (19.0%)	85 (35.4%)	0.43	0.12–1.22	0.145
**Tumour Related Factors**					
**Eloquent Tumour Location**					
No	8 (38.1%)	161 (67.1%)	Ref	-	-
Yes	13 (61.9%)	79 (32.9%)	3.35	1.36–8.78	0.010
**Operative Factors**					
**Anaesthetic**					
General	19 (90.5%)	145 (60.4%)	Ref	-	-
Sedation	2 (9.5%)	95 (39.6%)	0.58	0.09–2.13	0.480
**Adverse Event**					
No	19 (90.5%)	Ref	-	-	
Yes	2 (9.5%)	22 (9.2%)	1.05	0.16–3.96	0.947
**Post-operative Neurological Deficit**					
No	18 (85.7%)	215 (89.6%)	Ref	-	-
Yes	3 (14.3%)	25 (10.4%)	1.45	0.32–4.66	0.575

Readmitted cohort n=21; non-readmitted cohort n=240

OR = odds ratio; CI = confidence interval; Ref = reference category; WHO = World Health Organisation.

Odds ratios, confidence intervals and p-values are derived from univariable logistic regression analyses and relate to the 30-day readmission outcome

Interpretation should be undertaken with caution given the small number of readmission events (n=21) and limited statistical power

**Fig. 3:**
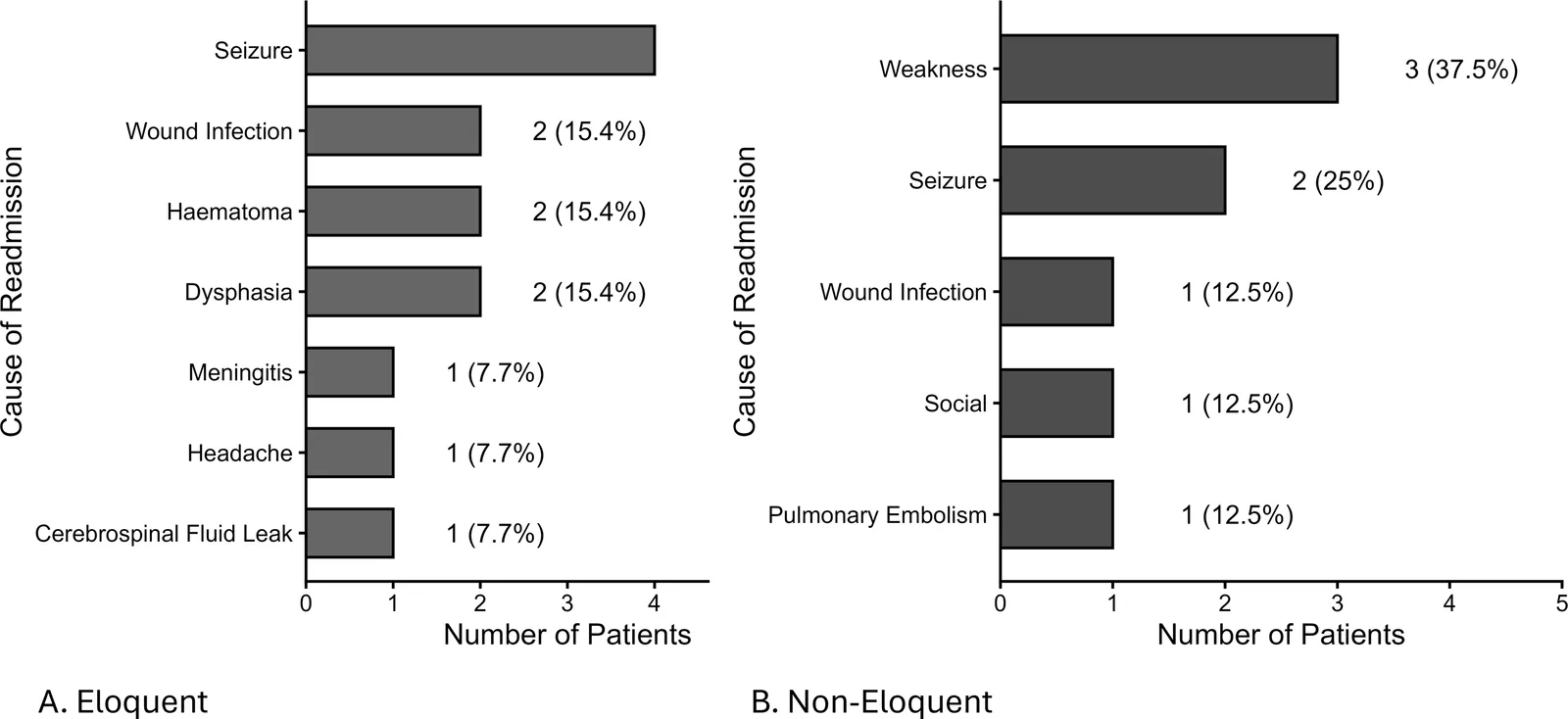
Causes of readmission in patients with eloquent and non-eloquent tumours. **A**. Distribution of readmission diagnoses among patients with tumours involving eloquent brain regions. **B**. Distribution of readmission diagnoses among patients with tumours located outside eloquent brain regions

Reasons for readmission within 30 days amongst those who had tumours in eloquent brain regions versus non-eloquent brain regions were compared (Fig. 3). The most frequent cause for the former group was seizures, whereas the latter mostly presented with weakness.

## Discussion

This study demonstrates that POD1 discharge following CTR is feasible in a substantial proportion of patients, with over half (55.5%) discharged on POD1. Importantly, early discharge was not associated with an increased risk of an unplanned 30-day readmission, supporting the feasibility of this approach within a structured discharge pathway.

These findings are in keeping with existing literature where selected neuro-oncology patients can be managed within an accelerated post-operative pathway without compromising clinical outcomes [1–3, 8]. Together, these findings suggest that early discharge was not associated with an observed increase in clinically relevant adverse outcomes in this cohort. However, the relatively small number of readmission events and wide confidence intervals should be considered when interpreting these findings.

The overall 30-day readmission rate of 8.0%, with a comparable rate of 7.5% among those discharged on POD1 indicates no increased risk of readmission associated with POD1 discharge. The causes of readmission were predominantly neurological in nature, reflecting recognised post-craniotomy complications rather than issues directly attributable to premature discharge. Causes for readmission among all patients were similar. It is worth noting that readmissions occurred across multiple regional hospitals, reflecting real-world patient pathways and supporting the applicability of a POD1 discharge pathway within a wider healthcare network.

Whilst readmission rate provides an important measure of post-discharge outcomes, cumulative 30-day hospital days may better reflect overall hospital utilisation. Patients discharged on POD1 had substantially fewer cumulative hospital days than those requiring prolonged admission (1.0 versus 7.0 days). This suggests that reductions in length of stay achieved through a POD1 discharge pathway were not offset by subsequent readmissions within 30 days.

Despite studies demonstrating an association between baseline functional status and length of stay, WHO Performance Status did not reach statistical significance in this cohort. This likely reflects pre-operative patient selection, as patients with poorer functional status are less likely to be considered suitable for accelerated recovery pathways. Therefore, the absence of an observed association should not be interpreted as a lack of clinical relevance, but rather as evidence of an appropriately selected surgical population [5–7].

Diabetes mellitus was the only pre-operative clinical factor that reached statistical significance and was associated with significantly lower odds of achieving POD1 discharge, suggesting that these patients may require more cautious peri-operative management to avoid prolonged admission. Despite the known impact of peri-operative corticosteroid use on glycaemic control, no association with discharge timing was observed. This suggests that corticosteroid administration alone should not preclude patients from consideration for POD1 discharge. Additionally, none of the patients who underwent unplanned readmission within 30 days were readmitted for diabetes-related complications. This suggests that the observed association may reflect clinical caution rather than increased diabetes-specific morbidity.

A higher WHO tumour grade and IDH wildtype status demonstrated significant associations with discharge timing. Each of these two variables showed an increased likelihood of POD1 discharge. The mechanism underlying this finding is unclear and may reflect unmeasured confounding or selection bias rather than a true biological effect. These findings should therefore be interpreted cautiously.

Post-operative factors demonstrated the strongest association with discharge outcomes, which is in line with existing literature [10]. The presence of a post-operative adverse event or neurological deficit was strongly associated with failure to achieve POD1 discharge. These findings emphasise that immediate post-operative recovery and neurological status are key determinants of discharge timing and reinforces the importance of structured inpatient monitoring in the early post-operative period within the frame of an early discharge pathway to ensure patient safety.

Baseline clinical factors showed limited predictive value of an unplanned 30-day readmission. However, eloquent tumour location was significantly associated with increased odds of readmission. Existing studies show resection within eloquent brain regions is associated with a higher risk of post-operative neurological deficits, which may necessitate prolonged recovery and rehabilitation [11–13]. These findings suggest that patients with tumours in eloquent brain regions remain at increased risk of delayed neurological deterioration following discharge, even in the absence of immediate post-operative complications. Targeted post-operative monitoring and support on discharge may help mitigate the risk of unplanned readmission in this subgroup.

Operational factors also contributed to discharge timing. Delayed post-operative MRI was uncommon, occurring in only five patients. Among patients without documented adverse events or neurological deficits, prolonged admission was predominantly related to completion of physiotherapy and occupational therapy assessment and clearance. These findings suggest that successful implementation of POD1 pathways depends not only on post-operative recovery but also on timely access to allied health services.

Interpretation of readmission outcomes should acknowledge the potential impact of selection bias. Patients discharged on POD1 represented a clinically selected subgroup who met predefined discharge criteria and demonstrated satisfactory post-operative recovery. Consequently, comparisons between POD1 and prolonged-admission cohorts should not be interpreted as demonstrating equivalence between discharge strategies. Rather, these findings suggest that structured POD1 discharge may be achievable in carefully selected patients without an observed increase in short-term readmission.

These findings have important implications for service delivery. The demonstration that POD1 discharge was feasible and was not associated with increased 30-day readmission supports wider implementation of structured early discharge pathways following CTR. A POD1 discharge pathway has the potential to reduce cumulative LOS and may improve bed availability, patient flow and healthcare efficiency within increasingly pressured healthcare systems. In the context of rising neuro-oncology caseloads, the ability to safely increase patient turnover without compromising outcomes is vital. Moreover, reduced inpatient stay is likely to confer potential economic benefits and could enhance patients’ quality of life by facilitating recovery within the home environment.

Several limitations should be acknowledged. As a retrospective study, it is subject to selection bias, as POD1 discharge was not randomly assigned. Only 21 readmission events occurred, limiting statistical power and resulting in wide confidence intervals around readmission estimates. Analyses were limited to univariable models and therefore do not account for potential confounding between clinical and tumour-related variables. Associations observed for WHO grade, IDH status and discharge outcomes should therefore be interpreted with caution, as residual confounding and selection bias may have influenced these findings. Also, reasons for prolonged admission were not prospectively recorded and therefore could not be analysed in detail. Finally, small subgroup sizes for several variables limited the precision of effect estimates.

## Conclusions

In conclusion, POD1 discharge following CTR was feasible in more than half of patients and was not associated with increased 30-day readmission. Post-operative neurological deficits and adverse events represented the principal barriers to achieving POD1 discharge, while eloquent tumour location was associated with subsequent readmission. These findings support the use of structured POD1 discharge pathways in appropriately selected patients. Larger multicentre studies are required to further evaluate factors associated with POD1 discharge and 30-day readmission. 

## Data Availability

No datasets were generated or analysed during the current study.
